# Iterative development of a pediatric point-of-care ultrasound training program

**DOI:** 10.1186/s12909-026-09444-9

**Published:** 2026-05-29

**Authors:** Aron Kerenyi, Albano de Juan Plaza, Serena Rovida, Simon Hansson, Karin Pukk Härenstam, Kathy Boutis

**Affiliations:** 1 Department of Women’s and Children’s Health, Karolinska Institutet, Tomtebodavägen 18A, 171 77 Solna, Sweden; 2 Department of Pediatric Emergency Care, Astrid Lindgren Children’s Hospital, Karolinska University Hospital, Stockholm, Sweden; 3 Center for Critical Care, University Health Network and Mount Sinai, Toronto, Canada; 4 Karolinska Institutet, Stockholm, Sweden; 5 Division of Pediatric Emergency Medicine, Department of Pediatrics, Hospital for Sick Children and University of Toronto, Toronto, Canada

**Keywords:** Pediatric POCUS, Curriculum development, Postgraduate training, Pediatric ultrasound

## Abstract

**Background:**

Point-of-care ultrasound (POCUS) is increasingly used across pediatric subspecialties. However, training opportunities in pediatric POCUS remain limited, and few studies have detailed the curriculum development process, making it challenging for other institutions to adopt. This paper describes the iterative development of a pediatric POCUS training curriculum in a setting without prior POCUS experience.

**Methods:**

We employed Kern’s 6-step approach to curriculum development to design a pediatric POCUS training program. The educational intervention was grounded in experiential learning and deliberate practice. A mixed-methods analysis of participant feedback was used for the iterative development of the course curriculum. Predictors of successful certification were analyzed using multiple logistic regression.

**Results:**

Between 2018 and 2023, 136 physicians participated in nine course iterations, providing 347 survey responses that informed course development. Overall satisfaction was high, but learners expressed a need for more supervised hands-on scanning with real patients than originally provided. The curriculum reached its final format after three iterations. Most in-person contact time was dedicated to supervised hands-on scanning. The inclusion of the online learning platform ImageSim enabled learners to develop image interpretation skills asynchronously. Learners participating in the final curriculum format and those who saved most scans initially were most likely to get certified. Costs were primarily associated with trainer salaries, which were covered by participation fees.

**Conclusions:**

Even in the absence of preexisting POCUS experience, our iterative curriculum development process led to the creation of a successful pediatric POCUS course in accordance with participants' needs. This study can serve as a guide for other institutions considering the development of a pediatric POCUS training program.

**Supplementary Information:**

The online version contains supplementary material available at 10.1186/s12909-026-09444-9.

## Background

Point-of-care ultrasound (POCUS) refers to the use of ultrasound at the patient’s bedside by the treating clinician who acquires, interprets, and integrates imaging findings into clinical care. Originating from the emergency department, POCUS has been integrated as part of routine practice in many clinical specialties, including but not limited to intensive care medicine, anesthesia, internal medicine, and obstetrics [1]. Within pediatric practice, pediatric emergency medicine (PEM) was the first to endorse POCUS as a core competency [2], followed by pediatric critical care, neonatology, pediatric hospital medicine, and most recently pediatric nephrology [3–5]. Consequently, POCUS is increasingly seen as a useful tool by both pediatric residents and general pediatricians [6].

However, training opportunities within pediatric POCUS are still lacking [6]. Few pediatric POCUS training programs have been described in the literature, and access to these programs in both North America and Europe is still limited [7, 8]. Additionally, the iterative nature of curriculum development is rarely described in the POCUS literature, which makes adoption by other institutions challenging. Thus, we describe the iterative development and evaluation of a pediatric point-of-care ultrasound training program.

## Methods

### Design and setting

In this report, we describe the pediatric POCUS program we developed between 2018 and 2023 at a university-affiliated urban pediatric tertiary care center. The program institution operates at two clinical sites, the main Pediatric Emergency Department (PED) being one of the largest in Scandinavia with a level I trauma center and a census of 60.000 visits annually. The POCUS program was implemented at the main PED due to its patient volume. There are approximately 60 pediatric residents who take regular shifts in the PED throughout their residency and 13 attendings, of which 8 are pediatricians and 5 are emergency physicians. Prior to the commencement of this training program, there was no POCUS use at our center.

### Pediatric point-of-care ultrasound course development

**Fig. 1:**
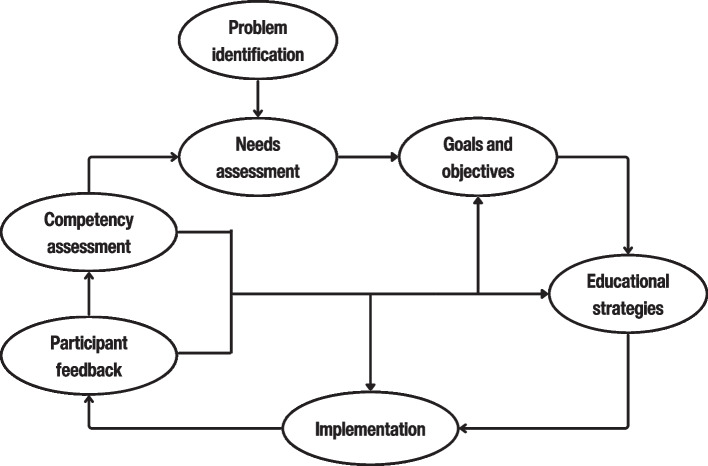
Application of Kern’s six-step curriculum development process in our study

In 2018, the PED leadership tasked two of the authors, a PED attending physician and a pediatric resident (AJP and AK) with forming a committee to create a pediatric POCUS course. The curriculum development followed the 6-step methodology described by Kern [9] in an iterative fashion [10]. This approach is a systematic curriculum development framework consisting of: (1) problem identification and general needs assessment, (2) targeted needs assessment, (3) goals and objectives, (4) educational strategies, (5) implementation, and (6) evaluation and feedback. We have used participant feedback together with certification outcomes and departmental POCUS use for refining the educational intervention (see Fig. 1).

### Problem identification and general needs assessment

The first step in our educational design was to analyze the current landscape and identify the need for a potential educational intervention. At the time, POCUS training was only available through external, two-day training programs without any focus on pediatrics. Participation in these training sessions did not lead to increased POCUS use among physicians at our site.

Furthermore, there were no published recommendations on the contents of a pediatric POCUS curriculum at the time.

### Targeted needs assessment and stakeholder engagement

Both the PED leadership and the attending group were recognized as keys stakeholders for the educational intervention. The course development committee utilized the PED attending group’s weekly meetings to informally gather information about their specific educational needs. Additionally, the committee had unscheduled meetings with the departmental and divisional leadership to make sure that the course development was conducted in accordance with the PED's strategic interests.

### Goals and objectives

The primary goal of curriculum development was to design a course that could be made available and relevant to all physicians in the Children’s Hospital as well as in other pediatric institutions in the country, given the overall lack of training opportunities in pediatric POCUS. To this end, the PED division head regularly discussed the course development with the director of the Children’s Hospital to ensure continued support. The course development committee also engaged other department heads to discuss curriculum content and multi-departmental course faculty (see Implementation). The course learning objective was defined as allowing participants to develop competency in acquiring, interpreting and integrating POCUS into their clinical practice, within the applications of their choosing.

### Educational theory

Competency in POCUS is a complex skill set, requiring the integration of theoretical knowledge, hands-on practice, and direct supervision with feedback [11]. Experiential learning theory, which emphasizes learning through concrete experiences followed by reflective observation, abstract conceptualization, and active experimentation, aligns well with these requirements [12]. This theory has been effectively applied in residency training and incorporates key elements essential for developing POCUS competency. Additionally, the framework of mastery learning with deliberate practice, which breaks down complex skills into manageable components for focused practice with immediate feedback, has proven successful in teaching POCUS image interpretation [13–15]. Therefore, our educational intervention was designed using a combination of experiential learning theory and deliberate practice principles. Both frameworks put a heavy emphasis on hands-on training and thus particularly rely on the availability of supervision which is addressed separately below.

### Educational strategies

The pediatric POCUS course was structured to be accessible to any physician treating pediatric patients in a hospital-based setting. Recognizing the varying relevance of clinical applications in different contexts, we adopted a modular design, allowing participants to tailor their learning to their specific needs. While the course was comprehensive, the primary focus remained on applications most relevant in the PED. Core content selection was guided by a comprehensive literature review and local practice priorities, resulting in the development of eight modules: one introductory and seven focused on clinical applications (Table 1).

**Table 1:** Outline of the prototype curriculum

Prototype Intervention Eight modules: • Introduction ◦ Physics ◦ Knobology ◦ Documentation • Lung ◦ Pneumothorax ◦ Pneumonia • Cardiac ◦ Focused Cardiac Ultrasound • Trauma ◦ Extended Focused Assessment with Sonography for Trauma (eFAST)	• Abdominal ◦ Intussusception ◦ Pyloric Stenosis ◦ Hernia • Urogenital ◦ Hydronephrosis ◦ Bladder volume ◦ Testicular • Soft tissue & Musculoskeletal ◦ Cellulitis/Abscess ◦ Foreign Body ◦ Hip ◦ Fracture • Procedural ◦ Peripheral IV access ◦ Regional Anesthesia

### Implementation

Participants prepared for each module by watching 60 mins of online instructional videos and completing a self-assessment questionnaire (see Supplementary Document 1) asynchronously, in a flipped-classroom format. The in-person modules were delivered in pairs over two-day blocks, during which participants engaged in a combination of lectures, case-based discussions, and supervised hands-on scanning sessions using participants as scanning mannequins. Additionally, children were recruited as model patients from the course faculty’s friends and family. Between these sessions, participants were expected to practice scanning during their clinical shifts, either under direct supervision or independently with indirect supervision. The faculty provided feedback on the saved ultrasound clips at least weekly, or within a day if specifically requested by participants, using published standards for POCUS image review [16]. Feedback was delivered either in person or via an online discussion forum (Slack Technologies, LLC, Vancouver, BC). The course took place within the span of 2–3 months, at the end of which participants could apply for certification in their chosen applications.

### Teaching faculty

A significant barrier to establishing a POCUS program is often the lack of access to skilled faculty for supervision [17], which was a challenge we also faced in implementing this curriculum. To address this, we engaged a POCUS expert from a different institution (SR), an internal medicine specialist with a Focused Ultrasound in Intensive Care certification, to supervise and participate in the prototype intervention. Additionally, we recruited ultrasound-trained teaching faculty from other departments, including pediatric anesthesia and intensive care and pediatric cardiology.

### Evaluation, feedback and redesign

Anonymous feedback was collected from participants at the end of each training day using a digital survey (Webropol Sverige AB, Sweden). This feedback included numeric ratings and free text responses related to the various educational activities (see Supplementary document 2). Descriptive statistics were used to summarize quantitative data while thematic analysis was employed to collate free text responses. Results were then shared with all relevant stakeholders. The teaching faculty held an evaluation meeting after each course iteration to discuss participant feedback and decide on adjustments to the course content and delivery format.

### Competency assessment

Participants received formative feedback throughout the course via self-assessment, direct and indirect supervision. Summative assessment was offered through an optional certification at the end of each course iteration; certification was voluntary as Swedish regulations do not mandate formal certification for POCUS practice. Additionally, learners from other regions would have had to arrange additional travel for the certification day which was rarely feasible and thus, this group was excluded from the certification analysis below. A critical-item checklist developed by Ma and colleagues for POCUS competency assessment was adapted to meet the curriculum’s requirements [18]. Univariable and multiple logistic regression was used to analyze predictors of certification success, including participant background (licensed physicians prior to residency, residents, PEM specialists, non-PEM specialists), course iteration (1, 2–3, and 4–9, reflecting major curriculum changes) and number of scans performed within two months of the course start (0–5, 6–10, and > 10 scans). Results are reported as odds ratios (ORs) with 95% confidence intervals (95% CIs).

## Results

### Course participation

**Fig. 2:**
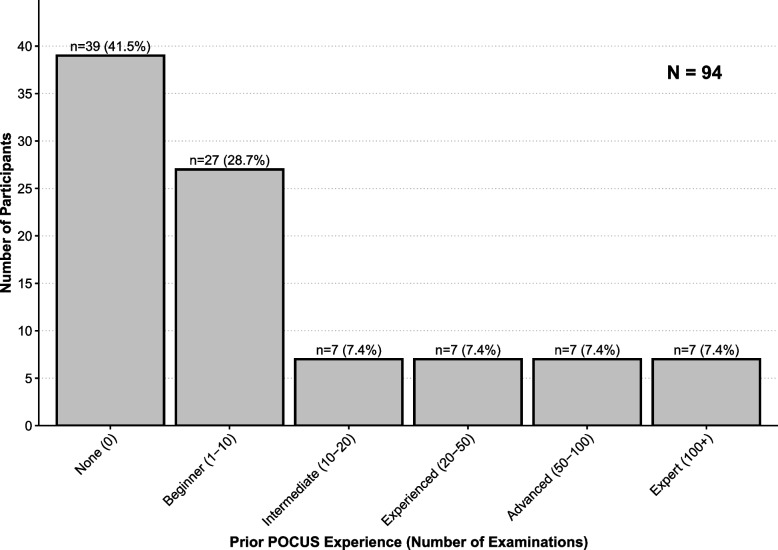
Participants’ self-reported prior experience with POCUS, collected via an anonymous survey at the start of the course

Nine iterations of the pediatric POCUS training program have been completed between 2018 and 2023 with a total of 136 participants. Participant characteristics are described in Table 2. Most participants reported no or little prior experience with POCUS (see Fig. 2). The largest group of participants were licensed physicians who were employed at the PED prior to starting residency. Residents accounted for 25% of the participants, mostly from general pediatrics. Attendings comprised 32% of course participants, the majority employed at the PED (41% of participating attendings) or at the Pediatric Intensive Care Unit (20% of participating attendings). Most participants were employed at the main hospital site where the course physically took place.

**Table 2:** Characteristics of course participants

Category	Group	*n* (%)
Training	Licensed Physician	54 (40%)
	Attending	44 (32%)
	Resident	34 (25%)
	Registered nurse	4 (3%)
Specialty	Pediatric emergency department	76 (56%)
	General Pediatrics	31 (23%)
	Pediatric intensive care unit	9 (7%)
	Emergency Medicine	6 (4%)
	Pediatric Nephrology	4 (3%)
	Extra corporeal membrane oxygenation	3 (2%)
	Neonatology	2 (1%)
	Pediatric Infectious Diseases	1 (1%)
	Pediatric Oncology / Coagulation	1 (1%)
	Pediatric Orthopedic Surgery	1 (1%)
	Pediatric Rheumatology	1 (1%)
	Pediatric Surgery	1 (1%)
Main Hospital	Main Hospital Site	107 (79%)
	Satellite Hospital Site	13 (10%)
	District General Hospital #1	10 (7%)
	Regional Hospital #1	3 (2%)
	District General Hospital #2	1 (1%)
	District General Hospital #3	1 (1%)
	Regional Hospital #2	1 (1%)

### Course evaluation

A total of 347 survey responses were received from the participants during the nine course iterations (Table 3). Numerical ratings were high for each of the nine course iterations (median overall rating 8.0–10.0 on a 0–10 scale), but qualitative evaluations contained many suggestions for improvements.

**Table 3:** Course evaluation summary. Median (IQR, min-max)

Course #	Course Content Median	Educational Methods Median	Course Material Median	Lecture Quality Median	Overall Summary Median
1	10 (0.00, 8–10)	8.0 (2.00, 4–10)	9 (2, 6–10)	10.0 (2.00, 8–10)	8.0 (2, 6–10)
2	10 (1.00, 7–10)	10.0 (2.00, 6–10)	9 (2, 6–10)	9.0 (1.00, 7–10)	9.0 (2, 6–10)
3	9 (1.00, 7–10)	9.0 (2.00, 5–10)	9 (2, 6–10)	9.5 (1.00, 7–10)	9.0 (1, 7–10)
4	10 (1.00, 5–10)	10.0 (1.00, 5–10)	10 (2, 5–10)	10.0 (0.50, 5–10)	9.5 (1, 6–10)
5	10 (0.25, 7–10)	10.0 (1.25, 6–10)	9 (2, 7–10)	10.0 (1.00, 6–10)	10.0 (2, 7–10)
6	10 (1.00, 7–10)	10.0 (1.00, 5–10)	9 (1, 5–10)	10.0 (1.00, 7–10)	9.0 (1, 7–10)
7	10 (0.00, 9–10)	10.0 (0.00, 9–10)	10 (1, 8–10)	10.0 (0.00, 9–10)	10.0 (1, 8–10)
8	10 (1.00, 7–10)	9.5 (2.75, 6–10)	9 (2, 6–10)	10.0 (1.00, 7–10)	9.0 (1, 6–10)
9	10 (1.25, 7–10)	9.0 (1.00, 7–10)	9 (2, 7–10)	10.0 (1.25, 5–10)	9.0 (2, 6–10)

Survey responses to quantitative summary questions about Course Content, Education Methods, Course Material, Lecture Quality and Overall Summary are presented for each course iteration. See Supplementary Document 2 for the full survey. Surveys during the prototype intervention used 1–6 scales which were normalized to 0–10 scales

Recurring themes in the qualitative evaluations of the first prototype curriculum included: more supervised hands-on scanning; more case-based discussions; shorter lectures and smaller groups. During the last six course iterations, which followed the redesigned curriculum described below, themes from participant feedback focused on improving the quality of the self-study material as well as improving the quality of supervision.

### Course revisions

**Fig. 3:**
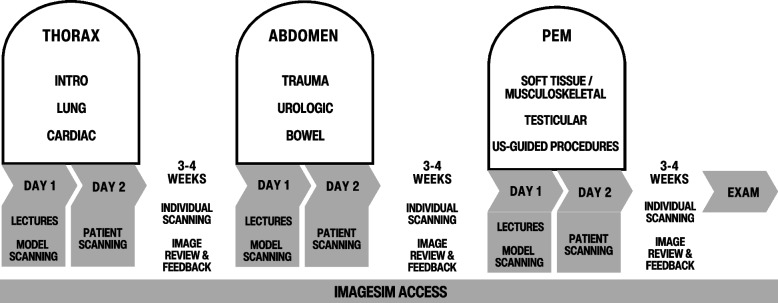
Outline of the final course design. PEM: Pediatric Emergency Medicine

Throughout the implementation period, the POCUS training program underwent iterative revisions based on participants’ feedback and evaluations from the teaching faculty. The course format was revised from eight one-day modules to three two-day modules to consolidate lecture content into three half-days and increase time for supervised scanning (see Fig. 3). Recruitment of healthy child volunteers as scanning models proved untenable in the long run. Instead, the second day of each module was dedicated to supervised scanning of pediatric patients at the PED and on the wards, with small groups of 3–4 learners per supervisor. This approach allowed learners to practice both scanning technique, image interpretation and clinical integration since many of these patients presented with pathological findings.

To provide access to more case-based self-directed learning, we reviewed available education solutions and chose to incorporate ImageSim, an image interpretation learning platform. Before applying for certification, learners were required to successfully complete the corresponding learning module on the ImageSim platform with 90% accuracy.

Due to a relatively low level of user engagement at the online discussion forum, this platform was replaced with a cloud-based image archiving software (Tricefy, Trice Imaging Sweden AB, Sweden). This HIPAA- and GDPR-compliant middleware system allowed for feedback and discussion between the teaching faculty and the participants side-by-side with the ultrasound clips.

Following the prototype intervention, no further course iterations required the participation of POCUS experts outside our institution. Although guest instructors from other pediatric departments were initially involved, the expansion of the POCUS course faculty at the PED to a team of six made it possible to conduct the training program independently. This reduced the scheduling challenges associated with coordinating support from other departments.

### Certification

Thirty-six of the 127 physician participants from our region (28%) applied for examination and 35 (97%) received certification in at least one module. Learners who participated in the final course format had an adjusted OR of 10.5 (95% CI 1.8–200.6) for getting certified compared to those who took part in the pilot curriculum. Learners who saved more than 10 scans within two months after starting the course had an adjusted OR of 4.5 (95% CI 1.4–16.0) for getting certified compared to learners who saved 1–5 scans. See Supplementary Table 1 for detailed results of the regression analysis.

### Impact on departmental ultrasound use

**Fig. 4:**
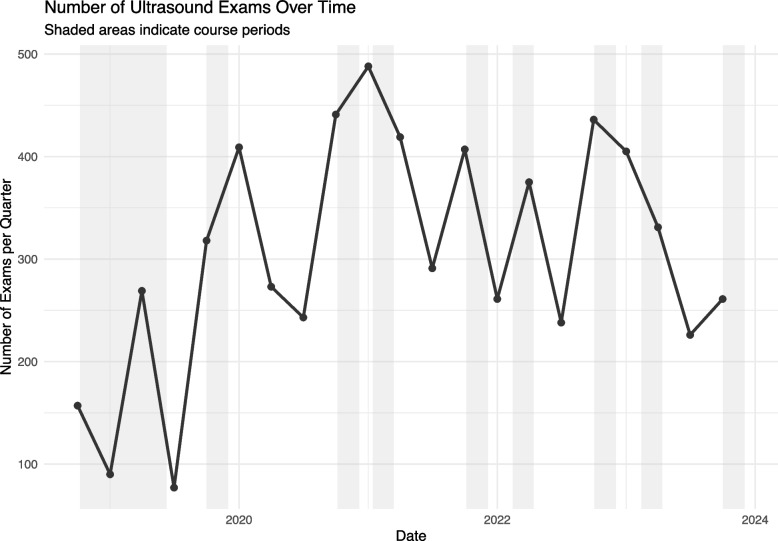
Quarterly number of ultrasound exams saved at the PED during the study period

The quarterly number of ultrasound exams saved in the PED has increased from 157 at the start to 305 at the end of the study period (see Fig. 4). Departmental ultrasound use was markedly increased during each course period.

### Costs

The pediatric POCUS course has been primarily financed using revenue generated from participant fees. The course venue and the ultrasound machines were available free of charge. The main expense has been the salaries of the teaching faculty, amounting to 26 person-days per course on average which translates to approximately 14.000 USD per course. We maximized the instructor-to-learner ratio at four students per instructor. The participation fee was set at the cost of approximately two faculty person-days (approximately 1,200 USD) which allowed for some margin for consumables and other costs. The new cloud-based PACS system and ImageSim POCUS courses [19] were financed by an innovation grant from the regional innovation office, but it may need to be considered in the future as an additional expense added to the participant fees.

## Discussion

The goal of this paper was to transparently describe the iterative development and implementation of a pediatric point-of-care ultrasound training curriculum which could be used as a blueprint by other centers to meet their needs of POCUS education. Participant feedback provided the basis for the gradual re-design of the educational intervention and the evaluation scores were very high throughout the implementation period. Most changes occurred during the first three course rounds, and the most significant feedback included more hands-on practice and case exposure. The curriculum has been stable for the past six iterations and continues to enjoy a steady stream of applicants from around the country. Overall, our experience demonstrates that even without extensive pre-existing POCUS expertise, it is possible to iteratively develop a pediatric POCUS training program with high levels of learner engagement alongside support at the departmental level. These findings are particularly relevant to healthcare systems where PEM is not a recognized subspeciality and POCUS is still relatively new.

Our experience demonstrates that participants learning this skill have a strong desire for in-person supervision of using POCUS at the bedside on real-life patients. While the prototype curriculum contained approximately 50% supervised scanning time using scanning mannequins, the overwhelming majority of participant feedback from this phase of development focused on the need for more supervised scanning time and real patient scenarios. Thus, the final curriculum reserved approximately 75% of the total contact time for supervised hands-on scanning: one third for scanning mannequins and two thirds for scanning actual patients. Similarly to our learners’ feedback, the lack of available supervision is repeatedly cited as a major limitation to adopting point-of-care ultrasound [17, 20]. At our site, we initially recruited experienced POCUS users from inside and outside our institution to fill this demand for in-person supervision. This aligns with the approach taken by the UK’s pediatric POCUS training program which allows practitioners holding non-pediatric ultrasound accreditation or those with significant experience with POCUS to serve as trainers in the Children’s Acute Ultrasound program [8]. Additionally, we encourage our most enthusiastic trainees to serve as junior trainers soon after getting certified, similarly to the UK’s training program. Therefore, we encourage all programs considering pediatric POCUS implementation to ensure the availability or capacity to build a cohort of faculty that provide in-person supervision.

Another challenge is providing learners with sufficient case exposure prior to independent practice. Bedside practice alone may not provide sufficient exposure to important but rare pathologies, especially within pediatric practice. While pathologies are presented during lectures and case reviews, these educational experiences are limited in time and don’t necessarily require active engagement from the learners. Thus, we opted to incorporate ImageSim, an evidence-based asynchronous educational platform into the course curriculum.

In brief, pediatric POCUS images are presented alongside a brief clinical stem and participants are asked to “make a call” on if pathology is present or absent. If present, they are required to locate the pathology. Participants receive text and visual feedback after every case and the goal is to strive for an evidence-based mastery learning standard [21], emphasizing deliberate practice [13, 15] and competency-based mastery [14]. This approach exposed participants to hundreds of cases over a few hours that included a breadth of normal and abnormal findings, building expertise in the image interpretation task which is thought to be one of the more challenging aspects of learning POCUS [13]. Including this type of training in our curriculum allowed more hands-on supervision during in-person contact time and created an opportunity for learners to develop high levels of image interpretation skills at their individual pace.

While there are no strict licensure requirements to practice POCUS in our setting, about 25% of our participants applied for certification. Participants from the revised curriculum were over 10 times more likely to get certified compared to participants from the pilot course, lending support for the curriculum development process. Additionally, participants who saved > 10 exams in the first two months after starting the course were several-fold more likely to get certified than participants with 1–5 saved exams. This finding underscores the central role of participant engagement in developing POCUS competency.

Importantly, our curriculum is not likely to have succeeded without ongoing support from PED leadership and affiliated departmental collaboration. POCUS applied by pediatricians can impact other specialties like intensive care, cardiology and radiology. Thus, optimal implementation should encourage collaboration between specialties since this can help with evolving trust and uptake of POCUS [22, 23]. Otherwise, the absence of collaboration may lead to other departments losing trust in the expansion of ultrasound use which can contribute to on-going friction between specialties [24]. We continually engaged with both Cardiology and Radiology departments via interdepartmental meetings. Collaboration between departments can take place at various levels, starting from recruiting participants and supervisors from multiple departments, as in our case, to conducting cross-specialty quality assurance, and to even higher levels of governance including a shared Enterprise Imaging Program [25].

Interdepartmental collaboration has been described as part of the curriculum development process in some published pediatric POCUS programs [8] but not others [7]. This discrepancy might be due to the different contexts these programs has been developed in – a number of PEM programs in North America have had an established POCUS curriculum for more than a decade whereas many European institutions are starting to develop these now. Future research could examine the models of inter-departmental collaboration that would optimize effectiveness and efficiency of implementing POCUS in a pediatric setting.

### Limitations

This was a descriptive study of a longitudinal curriculum development process which lacked some of the methodological rigor of prospective quality improvement studies. Our study was conducted in a single academic pediatric setting with funding to support the initiative and thus, our report may have limited generalizability to other sites or specialties. The costs associated with the course may limit the transferability of our results to resource-limited settings.

Additionally, participants self-selecting into taking this course and the lack of a comparison group may both pose a source of bias. Certification was not legally required for POCUS practice which may also introduce a selection bias into our analysis.

## Conclusions

Given the increasing recognition of POCUS as a core clinical skill across various pediatric subspecialties, the demand for pediatric-specific POCUS education is likely to grow. While workshops at conferences and other external training opportunities are valuable for introducing learners to POCUS, many institutions may find that in-house training is essential for establishing a system of supervision, accreditation, and quality assurance. We developed a pediatric point-of-care ultrasound training program using an iterative process based on the foundation of educational theories and participant feedback. Thus, this report can serve as a primer for other centers who consider establishing an in-house pediatric POCUS curriculum.

## Supplementary Information

Supplementary Material 1.

## Data Availability

The datasets generated and analyzed during the current study are not publicly available due to it constituting part of an internal quality improvement project but are available from the corresponding author on reasonable request.

## Abbreviations

PED: Pediatric Emergency Department
POCUS: Point-of-care ultrasound

## References

[1] Moore CL, Copel JA. Point-of-care ultrasonography. N Engl J Med. 2011;364(8):749–57. 21345104 10.1056/NEJMra0909487

[2] Marin JR, Lewiss RE. Point-of-care ultrasonography by pediatric emergency physicians. Policy statement. Ann Emerg Med. 2015;65(4):472–8. 25805037 10.1016/j.annemergmed.2015.01.028

[3] Singh Y, Tissot C, Fraga MV, et al. International evidence-based guidelines on point of care ultrasound (POCUS) for critically ill neonates and children issued by the POCUS Working Group of the European Society of Paediatric and Neonatal Intensive Care (ESPNIC). Crit Care. 2020;24(1):65. 32093763 10.1186/s13054-020-2787-9 PMC7041196

[4] Hopkins A, Doniger SJ. Point-of-care ultrasound for the pediatric hospitalist’s practice. Hosp Pediatr. 2019;9(9):707–18. 31405888 10.1542/hpeds.2018-0118

[5] Sethi SK, Mahan J, Hu J, et al. Point-of-Care Ultrasound (POCUS) Training Curriculum for Pediatric Nephrology: PCRRT-ICONIC Group Recommendations. Kidney360. 2024;5(5):671–80. 38477662 10.34067/KID.0000000000000415 PMC11146640

[6] McGinness A, Lin-Martore M, Addo N, et al. The unmet demand for point-of-care ultrasound among general pediatricians: a cross-sectional survey. BMC Med Educ. 2022;22(1):7. 34980087 10.1186/s12909-021-03072-1 PMC8722332

[7] Brant JA, Orsborn J, Good R, et al. Evaluating a longitudinal point-of-care-ultrasound (POCUS) curriculum for pediatric residents. BMC Med Educ. 2021;21(1):64. 33468138 10.1186/s12909-021-02488-z PMC7816421

[8] Griksaitis MJ, Zoica B, Raffaj D, et al. Development of the Children’s ACuTe UltraSound (CACTUS) point-of-care ultrasound (POCUS)-accredited training in the UK: a descriptive study. Arch Dis Child. 2024;109(7):543–9. 38442949 10.1136/archdischild-2024-326904

[9] Kern DE. Curriculum development for medical education : a six-step approach. Johns Hopkins University Press; 1998.

[10] Dolmans DH, Tigelaar D. Building bridges between theory and practice in medical education using a design-based research approach: AMEE Guide No. 60. Med Teach. 2012;34(1):1–10. 22250671 10.3109/0142159X.2011.595437

[11] Kwan C, Pusic M, Pecaric M, et al. The variable journey in learning to interpret pediatric point-of-care ultrasound images: a multicenter prospective cohort study. AEM Educ Train. 2020;4(2):111–22. 32313857 10.1002/aet2.10375 PMC7163207

[12] Kolb DA. Experiential learning : experience as the source of learning and development. Prentice-Hall; 1984.

[13] Kwan C, Weerdenburg K, Pusic M, et al. Learning pediatric point-of-care ultrasound: how many cases does mastery of image interpretation take? Pediatr Emerg Care. 2022;38(2):e849–55. 35100784 10.1097/PEC.0000000000002396

[14] Lee MS, Pusic MV, Camp M, et al. A target population derived method for developing a competency standard in radiograph interpretation. Teach Learn Med. 2022;34(2):167–77. 34000944 10.1080/10401334.2021.1907581

[15] Ericsson KA. Acquisition and maintenance of medical expertise: a perspective from the expert-performance approach with deliberate practice. Acad Med. 2015;90(11):1471–86. 26375267 10.1097/ACM.0000000000000939

[16] Physicians, A.C.o.E. Emergency Ultrasound Standard Reporting Guidelines. 2018 2025–11–03]; Available from: https://www.acep.org/siteassets/uploads/uploaded-files/acep/clinical-and-practice-management/policy-statements/information-papers/emergency-ultrasound-standard-reporting-guidelines---2018.pdf.

[17] Acuña J, Rubin M, Hahn B, et al. Point-of-care ultrasound in United States pediatric emergency medicine fellowship programs: The current state of practice and training. Pediatr Emerg Care. 2021;37(12):e1181–5. 32118834 10.1097/PEC.0000000000001955

[18] Ma IWY, Desy J, Woo MY, et al. Consensus-based expert development of critical items for direct observation of point-of-care ultrasound skills. J Grad Med Educ. 2020;12(2):176–84. 32322351 10.4300/JGME-D-19-00531.1 PMC7161337

[19] ImageSim POCUS Courses 2025–04–02]; Available from: https://imagesimcme.com/category/ultrasound.

[20] Meggitt A, Way DP, Iyer MS, et al. Residents’ perspective on need for point-of-care ultrasound education during pediatric residency. Hosp Pediatr. 2022;12(6):607–17. 35510494 10.1542/hpeds.2021-006444

[21] Cook DA, Brydges R, Zendejas B, et al. Mastery learning for health professionals using technology-enhanced simulation: a systematic review and meta-analysis. Acad Med. 2013;88(8):1178–86. 23807104 10.1097/ACM.0b013e31829a365d

[22] Handley MA, Gorukanti A, Cattamanchi A. Strategies for implementing implementation science: a methodological overview. Emerg Med J. 2016;33(9):660–4. 26893401 10.1136/emermed-2015-205461 PMC8011054

[23] Harel-Sterling M, Kwan C, Pirie J, et al. Competency standard derivation for point-of-care ultrasound image interpretation for emergency physicians. Ann Emerg Med. 2023;81(4):413–26. 36774204 10.1016/j.annemergmed.2022.11.002

[24] Olszynski P, Kim D, Liu R, et al. A multidisciplinary response to the Canadian association of radiologists’ point-of-care ultrasound position statement. Can Assoc Radiol J. 2020;71(2):136–7. 32063015 10.1177/0846537119898004

[25] Ma IWY, Francavilla ML, Nomura JT, et al. Governance considerations for point-of-care ultrasound: a HIMSS-SIIM enterprise imaging community whitepaper in collaboration with AIUM. J Imaging Inform Med. 2025;38(5):2585–99. 39753828 10.1007/s10278-024-01365-7 PMC12572525

