# Supplementary material for: Iterative development of a pediatric point-of-care ultrasound training program
Source: BMC Med Educ. 2026 May 29;26:874. doi: 10.1186/s12909-026-09444-9 (PMC13220600; doi:10.1186/s12909-026-09444-9)
Supplement: Supplementary file 1 — Supplementary Material 1. [file 12909_2026_9444_MOESM1_ESM.docx]

Supplementary Table 1 – Results of univariate and multivariate logistic regression predicting successful certification. Statistically significant results from the multivariate analysis are highlighted in bold font. OR: Odds ratio; aOR: adjusted odds ratio

| **Characteristic** | **Category** | **n = 127 (%)** | **OR** | **95% CI** | **p-value** | **aOR** | **95% CI2** | **p-value** |
| --- | --- | --- | --- | --- | --- | --- | --- | --- |
| Scans within 2 months | 1-5 | 60 (47%) | 1.00 | Ref |  | 1.00 |  |  |
|  | 6-10 | 26 (20%) | 4.00 | (1.23-13.69) | 0.022 | 2.92 | (0.81-10.99) | 0.103 |
|  | >10 | 41 (32%) | 7.77 | (2.87-23.79) | <0.001 | **4.49** | **(1.42-15.98)** | **0.014** |
| Participant category | Junior doctors | 54 (43%) | 1.00 | Ref |  | 1.00 | Ref |  |
|  | Residents | 32 (25%) | 0.56 | (0.19-1.49) | 0.261 | 0.67 | (0.21-2.04) | 0.484 |
|  | PEM specialists | 18 (14%) | 0.77 | (0.22-2.40) | 0.662 | 1.16 | (0.29-4.52) | 0.829 |
|  | non-PEM specialists | 23 (18%) | 0.30 | (0.06-1.02) | 0.078 | 0.64 | (0.12-2.90) | 0.572 |
| Course Iteration | 1 | 25 (20%) | 1.00 | Ref |  | 1.00 | Ref |  |
|  | 2-3 | 37 (29%) | 5.60 | (0.91-8.47) | 0.118 | 4.74 | (0.70-95.48) | 0.173 |
|  | 4-9 | 65 (51%) | 15.00 | (2.88-76.44) | 0.010 | **10.52** | **(1.83-200.55)** | **0.030** |

Supplementary document 1 – Example Self-evaluation MCQ test

**Lung ultrasound MCQ**

1. In a Pneumothorax study:
   1. Between two rib shadows, there is a notable echogenic line composed of the visceral and parietal pleura
   2. The transducer is placed longitudinally (pointed cranially) in the midclavicular line
   3. The transducer is moved inferiorly in a systematic fashion
   4. A and C
   5. A, B and C
2. The diaphragm is the hyperechoic line above the lever in the RUQ.
   1. True
   2. False
3. The ‘bat sign’ is:
   1. The artifact generated by the bowel when full of gas preventing the view of the abdominal aorta
   2. The anterior normal chest wall view with the probe placed longitudinally during pleura assessment
   3. The fractal interruption of the pleura surface observed in a lung inflammation process
   4. None of the above
4. Lung sliding: which of the following sentences is correct?
   1. Is pathognomonic sign of a pneumothorax
   2. Is made by B lines presence between 2 rib shadows
   3. Is ´to and fro´ movement of the pleura synchronized with the respiratory circle.
5. A-lines are:
   1. The reverberation artifacts generated by the interface between the ultrasound beam and air containing (eg normal lung) tissue.
   2. The artifacts seen in presence of air between two pleural layers.
   3. The air dots seen in the presence of lung inflammation.
6. A pneumothorax is investigated first in the:
   1. Inferior part of the lung when the patient is in a semi-recumbent position
   2. Superior part of the chest anteriorly in a semi-recumbent position
   3. Along the mid-axillary line
   4. None of the above
7. The artefacts defined as lines starting from the pleura layer and extending to the edge of the screen are known as:
   1. A lines
   2. E lines
   3. C lines
   4. B lines
8. The thymus in small children can be confused as:
   1. A pneumothorax
   2. A pneumonia in the anterior chest
   3. A pleural effusion
9. The absence of the lung sliding:
   1. Is sufficient to define a pneumothorax
   2. Is necessary for a pneumothorax diagnosis but not sufficient
   3. Is sufficient to define a pneumothorax in a trauma patient
   4. B and C
10. B lines mean:
    1. Interstitial syndrome (fluid between the alveoli)
    2. Asthma
    3. Pleural effusion

Supplementary document 2 – Course evaluation survey

**Self-study**

1. Have you watched the mandatory study video and taken the MCQ test?
   1. Yes
   2. No
   3. I dare not say
2. The self-study material was adequate in its breadth and depth, enabling satisfactory preparation for the course.
   1. 0 (Do not agree at all) to 10 (Completely agree)
3. What was missing from the material?

**Lectures:**

Lecture 1 (repeated for each lecture):

1. Presentation: The presentation was adequate and facilitated learning (interactivity, easy to follow, etc.):
   1. 0 (Do not agree at all) to 10 (Completely agree)
2. Content: The lecture content was of good quality (images, clips, text, other aids, etc.):
   1. 0 (Do not agree at all) to 10 (Completely agree)
3. Relevance: The lecture was relevant to the course's purpose:
   1. 0 (Do not agree at all) to 10 (Completely agree)
4. Suggestions for improvement:

**Summary of the day:**

Course Content:

1. The course content was relevant to the course's purpose:
   1. 0 (Do not agree at all) to 10 (Completely agree)
2. Which parts were the best during the training? Why?
3. Which parts can be improved? How?

Educational Methods:

1. The distribution between the different educational methods (theory, practice, group work, presentation) was well balanced.
   1. 0 (Do not agree at all) to 10 (Completely agree)
2. How can the course be changed to make it easier for participants to absorb the content?

Course Material:

1. The course material has been used in the best way during the course.
   1. 0 (Do not agree at all) to 10 (Completely agree)
2. How can the course material be improved?

Lecture Quality:

1. The lecturers have been pedagogical and structured in their presentations.
   1. 0 (Do not agree at all) to 10 (Completely agree)
2. Which lecturer/lecture was the least good?
3. Which parts can be improved? How?

Overall Summary:

1. What grade does today's course get?
   1. 0 (Fail) to 10 (Outstanding)
